# Risk management during times of health uncertainty in Spain: A qualitative analysis of ethical challenges

**DOI:** 10.1111/risa.17638

**Published:** 2024-08-30

**Authors:** Ignacio Macpherson, Juan J. Guardia, Isabel Morales, Belén Zárate, Ignasi Belda, Wendy R. Simon

**Affiliations:** ^1^ Bioethics Unit, Department of Humanities Universitat Internacional de Catalunya Sant Cugat del Vallès Barcelona Spain

**Keywords:** global common good, grounded theory, health uncertainty, humanistic skills, risk management

## Abstract

The study examines the reflections of various experts in risk management when asked about uncertainty generated by a health threat and the response to such a threat: what criteria should guide action when potential harm is anticipated, but not known with certainty? The objective of the research is to obtain a holistic perspective of ethical conflicts in risk management, based on experts’ accounts within the Spanish territory. A qualitative study was conducted through semi‐structured interviews with 27 experts from various fields related to health risk management and its ethical implications, following the grounded theory method. The method includes theory generation through an inductive approach, based on the identified categories. The 27 narratives obtained revealed a variety of fundamental issues grouped into 8 subcategories and subsequently grouped into three main categories. The first category focuses on human vulnerability in health matters. The second category explores the agents and instruments for decision‐making that arise from uncertain or traumatic social events. The third category refers to the need for common ethical paradigms for all humanity that implement justice over universal values. A main theory was suggested on the concept of responsibility in a global common good. There is an urgent need to assume this integrative responsibility as an inherent strategy in decision‐making. To achieve this, the involved actors must acquire specific humanistic training, conceptualizing fundamental ethical principles, and emphasizing skills more related to humanistic virtues than technical knowledge.

## 1 INTRODUCTION

The outbreak of the COVID‐19 pandemic in March 2020 caused profound global concern. This unprecedented phenomenon tested all healthcare systems worldwide (Munro et al., 2020). Since then, multiple studies have emerged addressing the ethical implications arising during the pandemic's spread. The list of these implications is extensive. Many relate to terminal illnesses (Constantinou et al., 2020; Tsamakis et al., 2020), some address dilemmas related to extreme sacrifice (Rasmussen & Dambrino, 2021; Riedel et al., 2022), others reflect on the issue of protective measures and people's beliefs (Moussaoui et al., 2020; Wald & Ruddy, 2021).

With the pandemic exposing the vulnerability of human nature—especially in the context of its interaction with the natural environment—the notions of “risk” and “uncertainty” have resurfaced strongly (Blaikie et al., 1994; Lee et al., 2023; Shi, 2016). Here, the term “risk” is understood broadly, as defined by United Nations Office for Disaster Risk Reduction (2015), where risk is “the probability of an outcome having a negative effect on people, systems or assets”. In turn, the concept of uncertainty is understood as the state, total or partial, of lack of information related to understanding or knowledge.

Until now, human intervention has been fairly successful in controlling or at least mitigating environmental threats, notably aided by technology (World Commission on Environment and Development [WCED], 1987). However, as Ulrich Beck (1992) noted in “Risk Society”, technological progress carries its own potential threats. It is evident that risks caused by humans are also numerous and serious. Some may pose a real threat to the environment and even human life (European Food Safety Authority Scientific Committee, 2019; Interagency Coordination Group on Antimicrobial Resistance, 2019; Luhmann, 2012; Yu & Kohane, 2019). In some cases, the damage is easily foreseeable, but in others, the damage is only probable, meaning there is no real certainty that the harm will occur.

In this context, the ability of humans to respond to a threat and its consequences becomes especially important. The limits and vulnerability of the natural world, as well as the limitations and vulnerabilities of humans, are being exposed and are concerning (International Bioethics Committee [IBC], 2013). This perspective raises a first question: what criteria should decision‐making follow when events with potentially uncertain harmful consequences occur? While it may seem that risk assessment at an individual level is adequately defined—for example, the implementation of informed consent—the same cannot be said for risks affecting society as a whole. These risks, often of an environmental or industrial nature, are more difficult to understand, let alone assume, and even more challenging to reverse (Kim et al., 2022; Krewski et al., 2014).

In many countries, this uncertainty (and the ethical dilemmas that it entails) have caused discrepancies in health management (Anderson et al., 2023; Malter et al., 2021; Montesó‐Curto et al., 2020). Health policymakers are increasingly forced to decide on issues where there is no scientific consensus (Rasmussen & Dambrino, 2021; Tsamakis et al., 2020; Wynne et al., 2020). Moreover, scientific uncertainty can take years or even decades to resolve. When human lives are at stake, such timelines are often not viable. Hence, complex yet urgent questions arise: what should be the accepted risk threshold for human life by society? What values will be compared? How to establish hierarchies among them? More specifically, should public authorities or companies be held responsible if their decisions prove harmful in the long run?

These questions compel us to question their protagonists, starting with representatives of the scientific community as well as those who will bear the risks through their initiatives (companies) and policies (public administration). Perhaps the most delicate area corresponds to the legal realm, due to the need to propose legislative changes that often affect fundamental rights. Finally, the opinion of decision‐making mechanisms represented by ethical committees becomes essential.

The above reflections frame the immediate objective of this research: to explore the ethical dilemmas generated by risk management in situations of uncertainty through a set of open questions addressed to a group of experts in the field and analyze their responses. The general objective is to develop a holistic perspective—a theory—that allows suggesting solutions to the ethical conflicts of risk management in situations of scientific uncertainty. The study focuses primarily on health, human safety, and the environment, with special emphasis on ethical considerations. In this sense, the integration of multidisciplinary knowledge is considered crucial to achieve this objective. Therefore, our starting point has been to value the opinions of experts from different areas of Spanish health management. Certainly, this is a study located in a specific territory, but we believe that the knowledge and experience of the experts are broad enough to provide a general perspective of this field. We consider that it can be illustrative and easily comparable with other societies (Arcos González et al., 2023; Montesó‐Curto et al., 2020).

## 2 METHOD

### 2.1 Study design

We conducted a qualitative study through semi‐structured interviews with experts from the Spanish territory belonging to areas related to health risk management and/or its ethical implications. The qualitative analysis followed the grounded theory method, which aims to generate theories to explain social phenomena (Eaves, 2001). It is an exploratory method, so there are no preconceived hypotheses as a starting point (Charmaz, 2006). Grounded theory thus allows contextualizing and understanding individuals’ subjective experience, applying a holistic approach to unknown situations. It is an inductive method that develops a theory based on individuals’ reality to explain a phenomenon. Some scholars recommend its use to describe human behavior in novel situations and generate substantive theories specific to an area (Barrow et al., 2023). Therefore, we consider it the most effective method to address the ethical dilemmas posed by experts in a situation of health‐related uncertainty.

The following steps were taken: a weighting of the central aspects of risk management to be addressed by the experts, formulated in the form of questions (Appendix); a selection of participants based on their knowledge of the study object; an exploration of participants’ narratives to identify the main reflections of the experts, up to saturation; a constant comparative method through coding, identifying categories and conceptualizations from the narratives; and the generation of a theory through the inductive approach, taking into account the identified categories.

The research protocol was submitted to Research Ethics Committee of the Universitat Internacional de Catalunya before the start of the study and was approved. All participating experts signed informed consent, agreeing to the use of their narratives in the analysis and publication of the study.

### 2.2 Participant selection

The sample population consisted of Spanish experts in healthcare risk management and knowledge of ethics and bioethics. This involved the participation of various professionals, both from the public and private sectors, including academics, businessmen, and politicians. The sample aimed to be representative, so the authors agreed to select experts from five different areas: (i) university professors of health sciences, (ii) university professors with experience in law, (iii) senior civil servants, (iv) highly qualified professionals in health‐related private companies, and (v) members of scientific and healthcare ethics committees.

Law experts were selected because we think that all risk management will involve the development and application of specific laws, without which no risk will be addressed by the executive power. Likewise, assuming a risk often involves the alteration of fundamental rights for society, making the intervention of lawmakers indispensable (Balbuena & Monaro, 2021; Rasmussen & Dambrino, 2021). The selection of healthcare experts was also considered essential as they provide the scientific objectivity implicit in all risk management. Furthermore, representatives from the business sector were included, as they are directly responsible for generating or resolving a significant portion of uncertainties associated with management. Representation from the public administration was also sought due to their connection with implemented policies and, in some ways, representing the State, ultimately responsible for management. Specifically, they are professionals holding high public positions related to the fields of health or the environment. Finally, the selection of members of ethics committees was deemed essential, as they are the usual decision‐making mechanism in risk management and responsible for advising on action policies in critical moments.

### 2.3 Data collection

In the initial phase of the research, eligible candidates were contacted via email. They were given a brief explanation of the study being conducted and invited to participate in an interview. Participants who agreed were sent an informational note about the interview methodology and an informed consent form. In this consent form, they were informed about their participation in the interview, its recording, coded transcription, and subsequent data analysis. Interviews were to be conducted in person and only exceptionally online. There would be no time restriction on answering the questions. The interviews were conducted in Spanish, and the informed consent stated that the statements would be used for analysis and dissemination of the research. The quoted texts presented in the article were translated into English by a native speaker to best express the idea conveyed in the transcribed narrative. Both the text of the informed consent and the interviews are stored by the authors for reference.

Collaboration was sought from 35 experts, out of which 27 agreed to participate in the research, 5 declined to participate, and 3 did not respond. The research concluded after conducting a total of 27 interviews: 7 law scholars, 4 health sciences scholars, 3 members of the Spanish Public Administration, 8 private enterprise professionals, and 5 scientific committee members. The semi‐structured interviews were conducted between November 2022 and May 2023 by all members of the research team, either online or in person.

### 2.4 Data analysis

Following data collection through semi‐structured interviews, an inductive analysis was conducted using the ATLAS.ti data analysis software, version 22.2.4. This program performed preliminary automatic coding, allowing for subsequent identification and coding of emerging themes from the narratives, aiding in the understanding of the interviews.

The analysis process began with data coding, grouping information into subcategories that were compared until identifiable patterns emerged. This coding was done in three phases: open, axial, and selective (Charmaz, 2006). In the open coding phase, different reflections were grouped into subcategories, a process carried out independently by two of the authors (IM_1_ and WS). Once these subcategories were determined, a second phase, axial coding, began, in which the previous subcategories were compared, grouped, and elaborated into complex categories. This process was conducted by two researchers (JG and IM_2_) and involving a third (IM_1_) in case of conflict.

Finally, in the third phase, selective coding was conducted, integrating the categories to reduce the number of concepts and thus delimit the theory underlying them all. The coding was performed by two authors (WS and JG) with the assistance of a third (IM_1_) for the final decision. During selective coding—the final and most abstract step of analysis—researchers identified the main concept of grounded theory. This concept helped explain the investigated phenomenon and connect all identified categories (Hallberg, 2006).

The interviews lasted approximately 50 min on average. Saturation was observed in 22 interviews. The remaining five were retained to confirm the data. Conversations revolved around central aspects of the research: identifying the most pressing current risks, addressing possible management strategies, and evaluating the role of ethics in risk management. During the open coding phase, interview responses generated eight subcategories. These subcategories corresponded to what experts perceived as most prominent when considering ethical dilemmas. Upon this structure, a second coding was elaborated. This new coding grouped the previous eight subcategories into deeper arguments, resulting in three emerging categories.

## 3 RESULTS

Below, the three categories are described along with the subcategories that each encompasses.

### 3.1 Category 1: assessment of health risks and their thresholds in a globalized society

Experts have explained what they consider to be the most pressing risks currently existing and the dimensions these risks may have. In addressing this issue, they have used the term “health risk” to refer to the likelihood of a health hazard occurring. Their responses were grouped into two main subcategories:

#### 3.1.1 Health/environment codependence

Experts agree that the primary cause for concern is human intervention, whether it be human interaction with one another or human interaction with nature. In their list of risks, there is a recurring concern regarding the following issues: climate change, zoonoses, food security, pollution, demographic dynamics, as well as those related to human behavior. These latter include mental health, addictions, certain novel lifestyles, challenges of reproductive technology, the use of artificial intelligence in decision‐making, and the sustainability of human systems: productive, healthcare, or financial.

I think that COVID has given us a good example that the intersection between human activity and nature is becoming more and more blurred, and the boundaries are becoming smaller or narrower, so that these threats are going to occur more and more (P2, law scholar)

However, experts point out a crucial aspect: the relationship many of these hazards have with each other. Climate change, food safety, diseases, soil pollution, inequalities, and impacts on abiotic (water, soil, and air) and biotic (fauna, landscapes, etc.) systems are interconnected.

There is a direct relationship between climate change and human diet or food safety. (…) Within these risks produced by climate change there is the affectation of public health, but above all on primary production, because primary production is directly affected by rainfall, pests, insects… (P1, private enterprise professional)

This paradigm leads to the belief that globalization has predictable effects. However, there may be many other effects of which we are still unsure. Therefore, some authors propose the need for global health or “One Health.” This concept considers the joint and global approach between three interconnected areas: human health, animal health, and the environment (WCED, 1987).

When we talk about ‘One Health,’ any medical decision should not only consider the patient, but it should not harm global health (P16, public administration member)

#### 3.1.2 Acceptance of human vulnerability

The previous section suggests a reflection linked to the interaction between human beings and the environment. All the experts who participated in the study shared the perception that the level of potential risks has increased exponentially and has caused a situation of permanent threat to the human being. Apparently, we are more vulnerable now than before. The extent of this threat became evident when COVID‐19 disrupted the dynamics of the entire planet. At this point, the consulted experts do not agree on the goal: if zero risk does not exist—since mere existence is already a risk—what level of safety should we expect?

In science and health, we always talk about the balance between risk and benefit. This is the threshold that has to be handled on both sides. There will never be zero risk, we never know what might happen (P22, private enterprise professional)

These questions force recognition of human vulnerability and adaptability as intrinsic principles to human beings.

Having a life expectancy of 90 years is not necessarily an indicator of health. For me, an indicator of health could be having a life expectancy of 80 years and, at the same time, fewer mental health diseases or a stronger sense of community (P17, ethics committee member)

Once they assume that there can be no zero risk, they necessarily resort to the use of statistics, the principle of proportionality, and the balance between harm and benefit to quantify that risk. Among the experts, there is a clear willingness to identify what the fundamental goods at stake are and to determine how to classify them optimally. Ultimately, determining risks involves establishing and prioritizing fundamental goods.

### 3.2 Category 2: decision‐making responsibilities

Once the experts have outlined the scope and persistence of the risk, they detail who should be responsible for its management and how it should be done. In this area, four recurring subcategories emerge in the interviews:

#### 3.2.1 Identification and coordination of agents

Experts identify several agents, but some appear periodically due to their special involvement to assume risks: scientists (for their diagnostic ability), private enterprises (for their capacity for action), and, above all, politicians (for their decision‐making capacity). Undoubtedly, other agents could be considered, although the experts do not point them out. All of these agents can demonstrate great professional competence, but, according to the experts, they will only be able to tackle the challenges if they demonstrate genuine excellence in social sensitivity.

When dealing with problems of social order it is absolutely necessary to have other inputs. Managers should have a humanistic training, apart from their specialty. Work on values, listening, empathic capacity, a certain capacity for social listening, where you are alert to what is happening in society (P24, public administration member)

Certainly, there are tools to coordinate the different agents in risk management, in the form of standards and preventive measures, inspection, and control, mainly in the environmental, health, and pharmaceutical sectors. They are the beginning and the basis of technical solutions.

We can mention a lot of legal tools that involve a development of a technical nature. In the Environmental Liability Act, manuals are designed, an inspection is carried out by competent persons to ensure that what is ordered is complied with (P10, ethics committee member)

But experts also agree on the insufficiency of these measures, expressing that the most effective means to resolve conflicts between the agents themselves is self‐regulation. This implies a capacity for dialogue and specific training whereby self‐regulation does not result in doing what each individual desires but deciding on something that can be accepted by others, even disregarding some of one's own considerations.

We are promoting self‐regulating codes. It seems to me that it is a good formula because, many times, the Government exercises a non‐binding leadership (P8, law scholar)

#### 3.2.2 Information transparency requirement and its extent

One of the responsibilities outlined by the experts is the obligation of transparency and information. They specifically value the quality and effectiveness of information over quantity or speculative information.

Regarding the degree of information, it is difficult to find a middle ground, but it seems to me that one errs more from silence than from wanting to create alarm (P6, health sciences scholar)

The goal is for authorities to generate trust and avoid states of widespread confusion through the transmission of truthful information at a progressive and tolerable pace. This is because society trusts in the activity of the managers and in their ethical behavior. In general, ethical parameters take precedence over statistical ones, and to achieve a balance between the good of society and transparency, there must be trust, a natural element of human beings and the foundation of their survival and development.

Ethics generates trust and generated a lot of peace in the colleagues of the work group. We never imposed, we gave our opinions, but we gave great importance to the ethical vision. They realized that ethics provided comfort for them, because it was the space we shared (P11, ethics committee member)

The way information is conveyed to the public is considered relevant as the transparency requirement itself. When providing information, the experts pointed out that the objectives should be (i) educating citizens, (ii) helping interpret data correctly, and at the same time, (iii) avoiding causing panic.

Depending on how a risk is communicated to you, you can clearly change. It is not the same to first say you have a 1% chance of dying or to be told that you have a 99% chance of living. Being the same information, by how they give it to you, you can act in one way or another (P3, law scholar)

#### 3.2.3 Decision‐making mechanism: referendum versus representative systems

Experts were asked about the effectiveness of the parliamentary representative system versus the referendum as a means to reach agreements during the decision‐making process under circumstances of scientific uncertainty. Undoubtedly, this would contribute to citizen participation in risk management, integrating individual risk and social risk. However, the results reflect clear skepticism regarding the referendum mechanism, although nonbinding surveys or informed consultations (such as in population segments or patient associations) are valued.

On the subject of a referendum, I am a little skeptical. Decision‐making, for example, on drugs that are prescribed to lower cholesterol cannot be advertised in generalist media (P20, ethics committee member)

The arguments of the experts have predominantly been based on the principles of responsibility, subsidiarity, and precaution. According to the experts, the existence of these principles, even if not very explicit, prevents falling into subjective conjectures, irrational panics, or benefit calculations that could bias decisions. In any case, this bias can also occur when selecting these principles by a group of experts. Hence the need to base decisions on the excellence of scientific and humanistic knowledge of decision‐makers, as well as on a certain level of trust in their decisions.

The population does not have the tools to challenge a referendum of this kind. Imagine all the difficulties that scientists have in responding to these questions (P27, private enterprise professional)

Regarding polling, all experts discard this potential form of direct democracy as a viable system for risk management. However, they do take it into consideration as a nonbinding tool for gauging the level of risk perceived by citizens.

I think that democracy should be representative, not direct. I think there has to be a delegation of responsibilities, in the sense that someone with the capacity and training can generate opinion (P9, public administration member)

#### 3.2.4 Accountability

Most experts agree that, in situations of uncertainty, the administration should assume ultimate responsibility for the outcomes. However, they also emphasize the need to consider (i) the conflict of interests at hand, (ii) the level of transparency exercised, (iii) the process of appointing decision‐makers, and (iv) who is accountable, in order to reach an agreement on the actions taken, taking into account all elements of the decision.

What good are declarations of principles if they do not commit the social partners, whether private or public? If there is no accountability, there is also no force for the issuance of its criteria (P19, law scholar)

Although there are already some mechanisms of accountability for certain areas of public administration, our experts agree on their limited enforcement in the strictly political sphere.

A number of accountability mechanisms already exist. I therefore think that we can use the ones we already have. But what we do have to understand is that, what we are not so used to is to evaluate the policies that are made. (…) A policy is executed and then rarely evaluated (P5, health sciences scholar)

### 3.3 Category 3: ethical foundations that shape the decision criteria

Most experts concur in the demand for a shared ethical foundation as a basis for action. However, experts recognize that their formulations on these ethical foundations do not seem sufficiently satisfactory to achieve that common goal.

#### 3.3.1 Decision‐makers’ key ethical criteria

Experts consider that the complexity of risk management arises from it seeming like a purely technical decision when, in reality, it is about resolving questions about what is good, bad, preferable, and dispensable. Often, risk management boils down to striking a balance between what we expect to gain and what we are willing to lose. To move forward, it becomes necessary to follow an ethical criterion to guide those decisions.

Risk involves deciding what is good and what is bad. Initially, a technical decision is made, studying all scientific notes. Then comes the moral part, knowing what is good and what is bad, and I have to resort to moral criteria. Either I find them in my community or I use moral autonomy (P12, law scholar)

Throughout the interviews, experts underline the need for a guiding principle, common to all, that can be taught and applied. Indirectly, the Universal Declaration of Human Rights of 1948 is widely appealed to. However, some experts attempt to define certain ethical paradigms to better fulfill that guiding role: the golden rule, non‐maleficence, human dignity, and the common good.

I think that we must first seek a common guiding principle. This principle must be possible, and it must be equitable. If you are going to do something that only benefits a sector of the population, you had better do nothing (P25, private enterprise professional).

Others appeal to more pragmatic criteria: social benefit, risk/benefit calculation, precaution, and cost of decisions. It is also not uncommon for some to resort to the exercise of virtue, especially prudence, when defining the universal ethical criterion.

We must maintain a dialogue of understanding, and this dialogue takes place in the ethical arena: it is the dialogue regarding values, virtues, principles, norms, rules that we have given ourselves. It's the Roussonian social contract we gave ourselves in the past (P10, ethics committee member)

#### 3.3.2 Responsibility toward future generations

Experts unanimously point out that the well‐being of future citizens is the responsibility of each member of present society. This duty is materialized in a broad concept of sustainability (health, financial, environmental, and psychosocial), understood as the capacity to meet the needs of the current generation without sacrificing the ability of future generations to meet their own needs, as outlined in the Brundtland Report (WCED, 1987).

We have obligations to future generations and that responsibility to the future. In a way, it is a responsibility that we already have with the present (P13, ethics committee member)

Experts acknowledge that it is difficult to foresee what the interests of future generations may be or what the needs of that world will be. However, the decision‐making criterion should be not to leave them a world in worse conditions than the ones we ourselves inherited.

There is a derivative risk, which has to do with the possibility of causing damage to future generations, and, above all, eliminating the capacity for action of future generations (P14, law scholar)

We see that acts of kindness that transcend oneself—even society itself—are considered virtuous acts. Furthermore, resorting to virtue, such as the generosity of exercising solidarity with the next generation, is considered a reference point for establishing responsibility in decision‐making. Thus, there emerges a need to revitalize the narrative around virtue, which entails the need to promote a humanistic education that deeply and rigorously analyzes these precise concepts.

Objective knowledge of reality leads to knowledge of ethical reality. There are some things that perfect the subject and others that degrade it. And it is inherent in the person to know that there are fundamental goods that must be protected and promoted (P21, health sciences scholar)

## 4 DISCUSSION

The narratives and viewpoints of this study have revealed a variety of existential issues, often associated with periods of crisis or catastrophes (Lee et al., 2021; Riedel et al., 2022), which may seem overly general for such a small area of study. As mentioned earlier, the study is limited to the point of view of Spanish experts, but the literature reveals a great deal of overlap with many studies conducted by other authors in a different context. We are aware that the literature on individual and social vulnerability requires further analysis (Borges et al., 2020; Wynne et al., 2020), but we think that our research can be considered a valuable starting point. We will now discuss each of these findings.

- Category 1, which we have formulated as “assessment of health risks and their thresholds,” highlights the vulnerability of human health, emphasizing the recognition and acceptance of human limitations and the imperative to safeguard human life and nature. This approach has been well analyzed by various authors (Blaikie et al., 1994; IBC, 2013; Monteverde, 2024). However, it seems important to us to emphasize the “health/environment codependence,” which gives rise to the concept of One Health and which can serve as a starting point for a change in attitude (Balkhy et al., 2018). As Graham and Wiener (1995) commented, the fragility of proposed solutions to prevent a risk can be discouraging, as one risk ends up generating another. Perhaps the cause is the absence of a global perspective on all of them. Therefore, only with a broad vision of health will we be, at least, more cautious when proposing solutions. This perspective connects with a paradigm that seems obvious: the “acceptance of human vulnerability,” as reflected in the Universal Declaration on Bioethics and Human Rights (United Nations Educational, Scientific and Cultural Organization [UNESCO], 2005). The analysis of existing risks and the acknowledgment of uncertainty have revealed the unavoidable vulnerability of humans to elements and to human interaction itself (Al‐Shammary & Hassan, 2023; Atwoli et al., 2022; Gambadauro et al., 2018). This perspective implies accepting the impossibility of knowing all elements of the problem, that is, we cannot know everything (Nilson & de Goër de Herve, 2023). But this conclusion does not block the ability to solve, although it does provide a good starting point, anchored in reality. From the recognition of one's own limits, society develops and internalizes the concept of responsibility, a key element in risk analysis. Hence, some authors consider vulnerability an intrinsic principle of human beings: humans become human thanks to their vulnerability (Kottow, 2004; Rendtorff, 2002; Ten Have, 2014).
- Category 2 emphasizes “decision‐making responsibilities,” related to uncertain events in society: the need for more agile and transparent communication, the demand for cooperation among agents, as well as the emphasis on accountability in decision‐making. Responses to this vulnerability that have been carried out over time have provided us with some essential values. These are already largely present in predominant ideologies and religions (Unger et al., 2020) and have recently been tested by risky circumstances (Parisi et al., 2021; Wieringa et al., 2023). Hence, the importance of “identifying and coordinating the responsible agents” for management. Authors agree on their identification but differ in their coordination and the order of responsibilities (Barrow et al., 2023; Morgan et al., 2022; Wald & Ruddy, 2021). In this regard, there is a moderate consensus in determining the most effective means of initiating conflict resolution: self‐regulation and the risk‐sharing models, but that does not imply diluting the responsibility of science, business, or politics (Blumenthal‐Barby et al., 2019; Hill et al, 2018). Likewise, the “information transparency requirement” emerges as a key element, where the need to be informed constitutes a human right, widely accepted (Gochfeld, 2022; World Medical Association [WMA], 2013; Wynne et al., 2020). The same cannot be said regarding the “decision‐making mechanism,” where there is no clear agreement. The use of referendums as a decision‐making mechanism, reflected in this subcategory, is not evident in the literature, and many authors express reservations (Ho et al., 2014; Lachapelle et al., 2021; Perrella & Kiss, 2015). The most common trend is to resort to the leadership of individuals and entities (Jonas, 1985; Andorno, 2004; Beskow et al., 2018). Finally, the instrument that garners the most agreement is presented in the “accountability” subcategory. Here, the conflict of interests, transparency, determination of decision‐makers, and assumption of blame are evaluated and discussed by various authors (Blumenthal‐Barby et al., 2019; Gochfeld, 2022; Malter et al., 2021; Morgan et al., 2022).
- Category 3 addresses the “ethical foundations that shape the decision criteria.” Expert reflections conclude that risk managers (scientists, entrepreneurs, and politicians) play a leadership role in these situations but suggest the need to reinforce their humanistic preparation (Stetson et al., 2020; Wald & Ruddy, 2021). Therefore, in developing the “decision‐makers’ key ethical criteria,” expert proposals lean heavily toward metatechnical solutions: increasing the exchange of experiences (Morgan et al., 2022), becoming more resilient to challenges (Kok et al., 2023), and promoting global altruism (Armstrong, 2006; Maci & Marešová, 2022). Following the experience of the COVID‐19 pandemic, experts agree on appealing to ethics. Ethics engenders a higher level of trust in the population than relying solely on science (Frewer et al., 1996). Therefore, although humanistic solutions sometimes involve fuzzy concepts, society more readily assimilates them than purely scientific solutions. Likewise, “responsibility toward future generations” is conceptualized as a moral obligation, not pragmatic. Thus, there is repeated, perhaps utopian, appeal to the need for a common ethical paradigm for all humanity as a guide for directing efforts toward universal values. This would include prioritizing the common good over individual good and the need for individual and collective commitment to survival (Glückstad et al., 2021; Unger et al., 2020).

Based on these three categories, we outline a theory that can integrate these reflections, deducing the essential principle that guides decision‐making in human beings: it is not their purely technical capacity but their ability to unconditionally support each other (de Goër de Herve et al., 2023; Kabir et al., 2021). We argue that this trait is similar to Aristotle's concept of benevolent friendship, which understands benevolence as “willing the good of another” (Aristotle, 2014). It is a concept that all societies seem to take for granted, albeit somewhat thoughtlessly (MacIntyre, 2007). This concept of benevolence is the criterion underlying distributive justice when it comes to the allocation of scarce resources; prioritizing the common good over individual good; the need for freedom to be directed toward the good and not merely the opening of new options; solidarity in the face of irresolvable conflicts; the balance between vulnerability and the human capacity for transformation (Nacoti, 2020; Glückstad et al., 2021).

**FIGURE 1:**
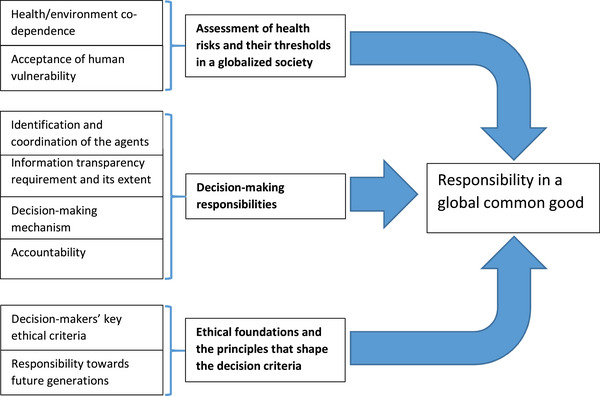
Conceptual model of global common good theory.

The theory we propose, outlined in Figure 1, indicates that vulnerability (especially health‐related vulnerability) has globalized to such an extent that technical or scientific responses are no longer capable of addressing it. Consequently, this vulnerability requires global responsibility, based on a concept of common good that transcends any sociopolitical barrier.

It is necessary to clarify the concept of common good. It is a concept that is observed in various cultures, outlined in the theories of Plato and Aristotle, and subsequently developed by various authors, such as Thomas Aquinas, John Locke, Jean‐Jacques Rousseau, John Stuart Mill, or John Rawls. In this sense, it would be the set of conditions of social life that make possible, for each of its members, the fullest achievement of one's own perfection; it derives from the human condition and is therefore superior to any individual; it benefits everyone and encompasses the entire person, that is, both the demands of the body and those of the mind; it obliges the state and obliges individuals, in space—the whole human society—and in time—future generations (Rawls, 1999).

In this context, risk analysis constitutes a realistic and effective way to promote this common good, since risks point to factors that affect the assets of the person and society as a whole. Most of the risks indicated in this study refer to safety and health, which are the most basic goods of people, but the global common good suggests others that are essential to configure a full life, such as the right to privacy, the right to education, equality before the law, or equal opportunities. The main objective, as Ricoeur (1992) expressed it, would be a good life with and for others within just institutions. In this sense, the ethics of the global common good considers that the common good is much more than the sum of its parts and exceeds the limits of the individual.

The theory constitutes a call to responsibility for a global common good in the face of increasingly globalized risks through a new perspective on decision‐making agents (Jonas, 1985; Morgan et al., 2022; Rockström et al., 2024): the perspective of the intrinsic benevolence of all human decisions. This perspective must necessarily rest on competent humanistic education (Wald & Ruddy, 2021), capable of discerning common ethical principles, more rooted in the exercise of virtue than in pure scientific knowledge (UNESCO, 2005; WMA, 2013).

## 5 STRENGTHS AND LIMITATIONS

A primary strength of this study is having had the opportunity to analyze the extensive and freely expressed opinions of experts, some of whom are agents directly involved in decision‐making within our research area. Second, the academic composition of the research team itself is a strength, as it includes researchers from all fields involved in the scrutinized topic: technology, philosophy, science, politics, economics, and law.

The main limitation of this research is the subjectivity of the qualitative method, which requires objectifying narratives into study objects. This has been attempted by means of reflective rereadings of the material by team members in pairs and with arbitration in cases of doubt.

It is also a limitation to have restricted the scope of the study solely to the opinions of experts, thereby omitting the valuable contribution, among others, of a selection of vulnerable populations that have been more directly affected by the situation of risk and uncertainty. Undoubtedly, this may have biased the information, but we think that a wide variety of areas are covered to deduce conclusions. We understand that these should be taken into account for future research.

And finally, we mention the existence of bias when selecting exclusively Spanish experts, but we considered that it was relevant to know their points of view on global issues and that they have responded as knowledgeable about the reality of the planet. Certainly, some aspects discussed depend greatly on national structures, especially those related to legislation and politics. The public administrations of each nation react differently to uncertainty, a divergence that is less common in health or scientific committees, in which the criteria for action are similar. The most notable differences have to do with methods of political decision‐making, transparency, or accountability, in which countries differ according to their cultures. For this reason, an attempt has been made to address the problems from a global perspective, so that the similarity of the problems and the common principles that underpin the solutions are evident (Beck, 1992; Luhmann, 2012).

## 6 CONCLUSIONS

The conclusion that emerges from the analysis of the experts’ reflections is the need to assume responsibility for a global common good, understanding the common good as the set of conditions that make possible, for each of its members, the fullest achievement of one's own perfection. This responsibility must be an inherent strategy in making decisions about risk management in situations of health uncertainty. For this, it is essential that the agents involved in risk management have specific humanistic training. Deficient or merely technical training in this field can be detrimental to essential aspects of individual and collective human existence. The first phase of this training should consist of a proper conceptualization of fundamental ethical principles common to all societies, as well as the implementation of skills more linked to humanistic virtue than to technical ability. We consider the reflections of experts and their analysis to be a suitable starting point for a more detailed exploration of the reasons that drive human action in moments of risk.

## CONFLICT OF INTEREST STATEMENT

The authors declare no conflicts of interest.

## ISSUES

1. In your opinion, what are the most relevant risks today and what bioethical dilemmas do they present?
2. How to determine the acceptable risk threshold for society?
3. How should risk management be coordinated between all social agents (administration, scientific committees, companies, academics, and civil society)? Should it be done by consensus or should agreements be reached in another way?
4. Given new studies that call into question the safety of a substance, what should be the criteria for action?
5. What would be the level of information that should be conveyed to the public regarding the results of risk analysis so that they generate a sense of trust? Should it include the reports of the social agents (committees, administrations, companies, etc.)?
6. If greater participation was provided to society—for example, in the form of a referendum—do you think the risks would be managed better?
7. What ways would society's behavior in the face of risk be regulated?
8. Should the social agents involved (administrations, committees, companies, etc.) be held accountable in risk management and how could this be articulated?
9. What should be the fundamental ethical criterion for decision‐making in risk management?
10. In situations of uncertainty, what could be the ethical criteria of the social agents involved (committees of experts, administrations, companies, etc.)?
11. Is it possible to determine ethical duties with future generations? How?
